# Supplementary material for: Ubiquitin and a charged loop regulate the ubiquitin E3 ligase activity of Ark2C
Source: Nat Commun. 2022 Mar 4;13:1181. doi: 10.1038/s41467-022-28782-y (PMC8897509; doi:10.1038/s41467-022-28782-y)
Supplement: Supplementary file 1 — Supplementary Information [file 41467_2022_28782_MOESM1_ESM.pdf]

Supplementary Figures

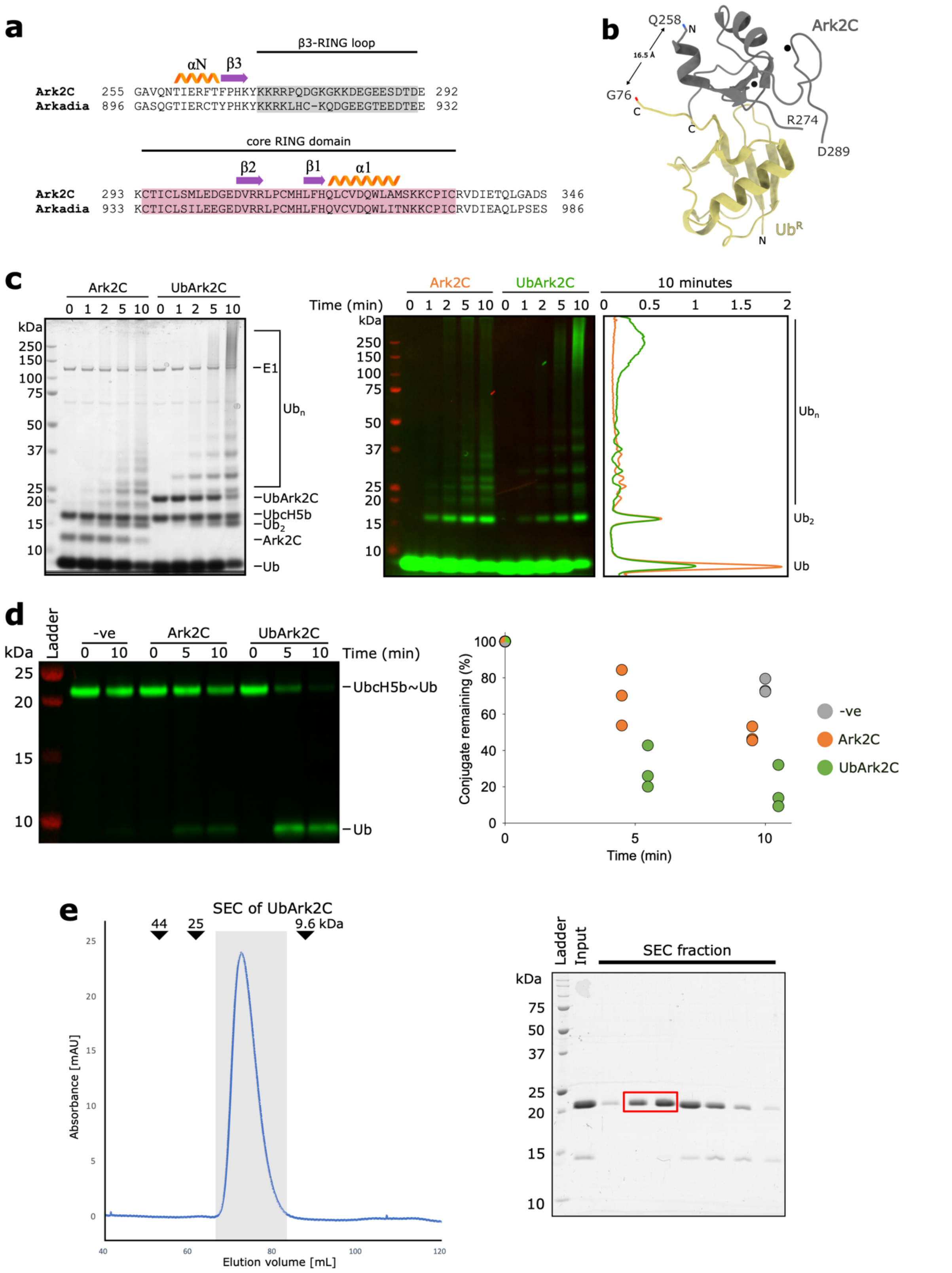

Supplementary Fig. 1 *Design and analysis of the UbArk2C fusion protein*. (a) Sequence alignment of Ark2C (255-346) with Arkadia isoform 2 (896-986), highlighting the β3-RING

loop, core RING domain and the elements of secondary structure. Note β1 and β2 are designated as shown to be consistent with other RINGs. (b) The Ark2C-Ub^R (grey-yellow) complex reported previously is shown as a cartoon with zinc ions depicted as black spheres (PDB: 5D0K). The C-terminal oxygen of Ub^R (Gly76) and the N-terminal nitrogen of Ark2C (Gln258) are depicted as sticks, with the distance between them shown. (c) Multiturnover ubiquitylation assays comparing the activity of Ark2C and UbArk2C. The gel on the left was stained with Coomassie Blue. Cy3-labelled ubiquitin was included in the assay and the same gel was imaged using fluorescence and Image Studio Lite (right). To enable comparison the intensity of Cy3-labelled ubiquitin in the 10 minute timepoint was compared by linescans (Ark2C in orange and UbArk2C in green) and is shown on the far right. Intensity (arbitrary units) is plotted against distance migrated. (d) Single-turnover assay comparing the release of Cy3-labelled ubiquitin from UbcH5b~Ub following addition of Ark2C and UbArk2C. The assay was performed in triplicate using 0.55 µM E3 ligase and 4 mM L-Lysine, and the quantified data is shown to the right. Source data are provided in the Source data file. (e) The SEC elution profile for UbArk2C is shown and the position of molecular weight standards (Ovalbumin 44 kDa, Chymotrypsin 25 kDa, and His_6-Ub 9.6 kDa) is indicated. Fractions from across the peaks are shown at right. The red box indicates fractions combined for crystallisation.

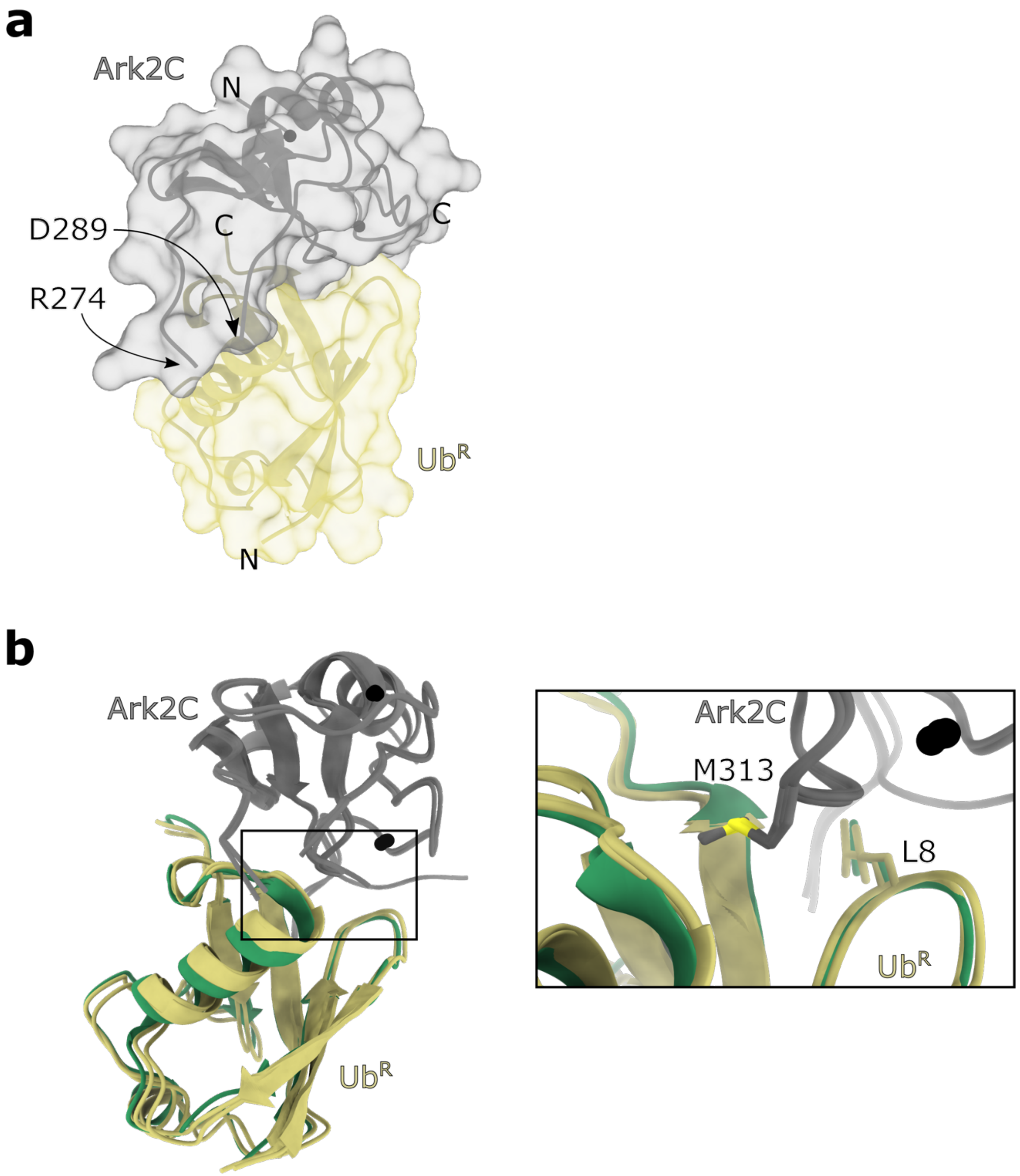

Supplementary Fig. 2 *The crystal structure of UbArk2C*. (a) The structure of the UbArk2C fusion is shown as a semi-transparent surface. Ub^R is shown in yellow and Ark2C in grey. (b) Comparison of Ark2C-Ub^R complexes. The overlay was prepared by aligning the Ark2C molecules from both copies of the UbArk2C structure (yellow) and one copy from the Ub-Ark2C complex reported previously (PDB: 5D0K, green).

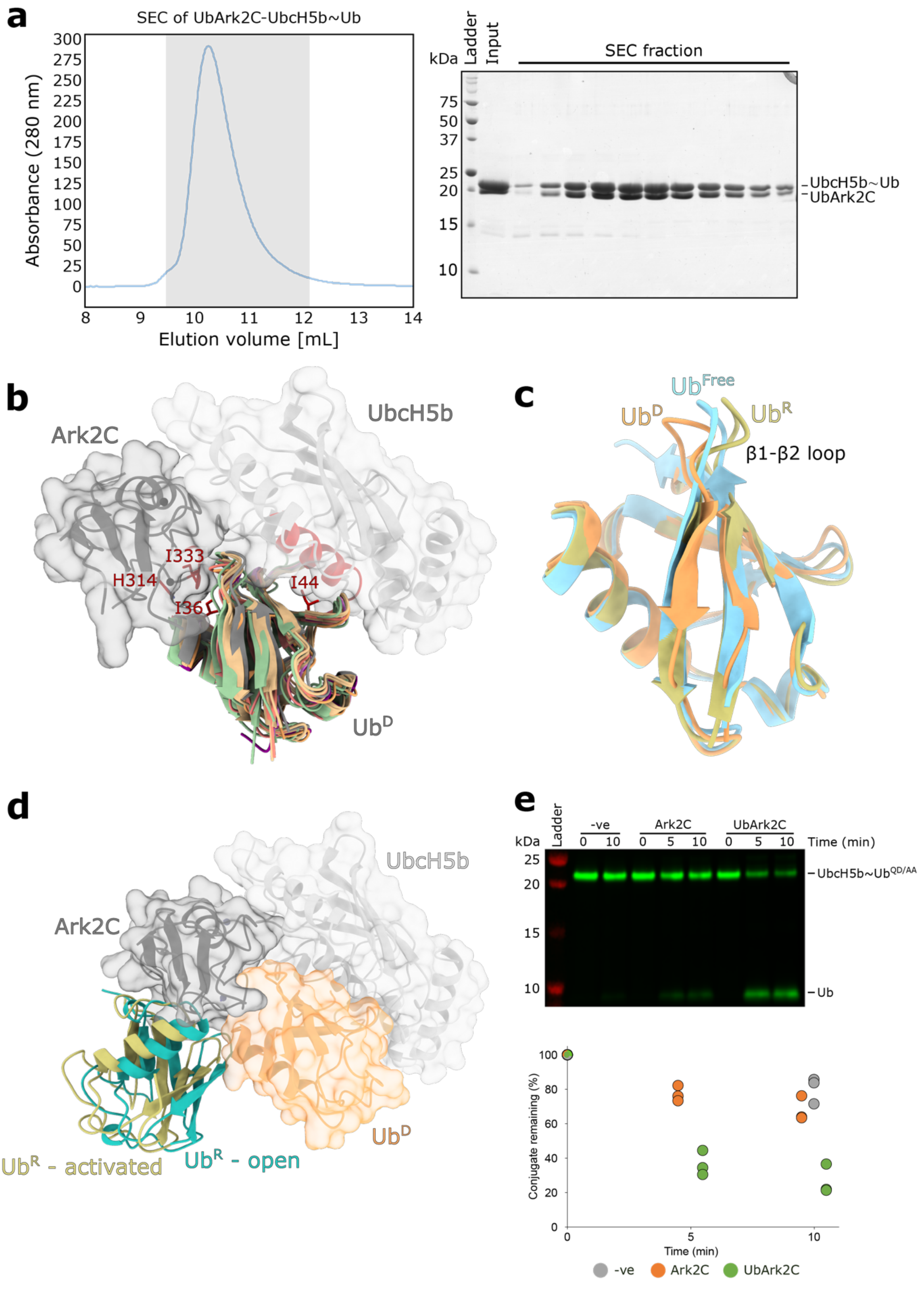

Supplementary Fig. 3 *The crystal structure of the UbArk2C-UbcH5b~Ub complex.* (a) UbArk2C and the isopeptide-linked UbcH5b-Ub conjugate were copurified by SEC. The fractions (right) indicated a 1:1 complex formed. (b) Overlay of the UbArk2C-UbcH5b-Ub complex with the

following E3s: TRAF6, cIAP1, LNX1, MDM2:MDMX, TRIM25, RNF4, Cbl-B, RNF38, BIRC7 and MDM2, TRIM21 (PDB: 5VO0, 6HPR, 5H7S, 5MNJ, 5EYA, 4AP4, 3ZNI, 4V3L, 4AUQ, 6SQS, and 6S53) closed conformation E2~Ub conjugate complexes. Only Ub^D from these complexes is shown and the conserved position of Ile44 and Ile36, which contact the crossover helix in the E2 and the RING domain respectively, are shown. (c) Overlay of Ub from UbcH5b~Ub (orange) and from the UbArk2C fusion (green) in the Ark2C closed conformation complex, as well as free Ub (blue) (PDB: 1UBQ), showing the orientation of the β1-β2 loop. (d) The structures of UbArk2C and the UbArk2C-UbcH5b~Ub complex were overlayed using Ark2C. Just Ub^R (cyan) from the UbArk2C structure is shown to highlight its displacement to accommodate Ub^D in the complex. Molecules are coloured as before: Ark2C (dark grey), UbcH5b (pale grey), Ub^R (yellow) and Ub^D (orange). (e) Single-turnover assay comparing the release of Cy3-labelled ubiquitin from UbcH5b~$Ub^{QD/AA}$ by Ark2C (orange) and UbArk2C (green). The assay was performed in triplicate using 0.7 µM E3 ligase and 2 mM L-Lysine, and the quantified data is shown below. Source data are provided in the Source data file.

a

Monomer1	Monomer2	Symmetry operation	Area [Å^2]	DeltaG [kcal/mol]	H-bonds	Salt bridges
UbcH5b	UbcH5b	X,-Y-1,-Z	603.9	-6.3	2	0
UbcH5b	Ub^R	X,-Y,-Z	374.7	-1.7	0	3
UbcH5b	Ub^R	-X-1,-Y-1/2,Z-1/2	305.3	-0.8	2	2
UbcH5b	Ub^R	-X,-Y-1/2,Z-1/2	203	-1.7	1	0
Ark2C	Ub^R	-X,Y-1/2,-Z+1/2	183.4	1.4	3	0
UbcH5b	Ark2C	-X,-Y-1/2,Z-1/2	152.8	2.1	3	0
UbcH5b	Ub^D	-X-1,-Y-1/2,Z-1/2	105.9	0	0	0
Ub^D	UbcH5b	X-1,Y,Z	45.1	-0.6	0	0
Ub^D	Ark2C	X-1,Y,Z	24.8	0.2	0	0
Ark2C	Ark2C	X,-Y,-Z	24.2	0.5	0	0
Ub^D	Ub^R	-X-1,Y-1/2,-Z+1/2	18.2	1	0	0
UbcH5b	Ark2C	-X-1,-Y-1/2,Z-1/2	12.9	0.6	0	0
UbcH5b	Ark2C	X,-Y,-Z	6.7	-0.1	0	0
UbcH5b	Ark2C	X,-Y-1,-Z	6.4	0	0	0
UbcH5b	Ub^R	-X,Y-1/2,-Z+1/2	4.2	0	0	0
Ub^D	Ub^R	X,-Y,-Z	3.4	0	0	0

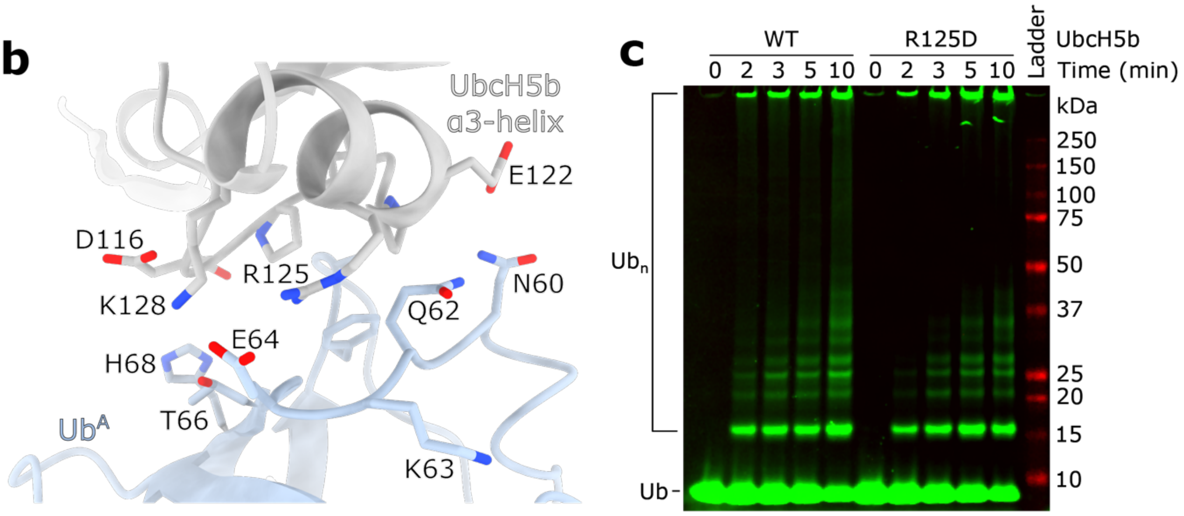

Supplementary Fig. 4 *The catalytic complex.* (a) Table of crystal contacts generated by analysing UbArk2C-UbcH5b~Ub complex using PISA. Only the interfaces outside the crystallised complex are shown. The potential UbcH5b-Ub^A contact is highlighted. (b) The crystal contacts between UbcH5b and Ub^A from the catalytic complex are shown. (c) Multiturnover assay comparing the activity of WT UbcH5b and UbcH5b R125D. The assay was performed using Cy3-labelled ubiquitin and the gel was imaged using fluorescence and Image Studio Lite.

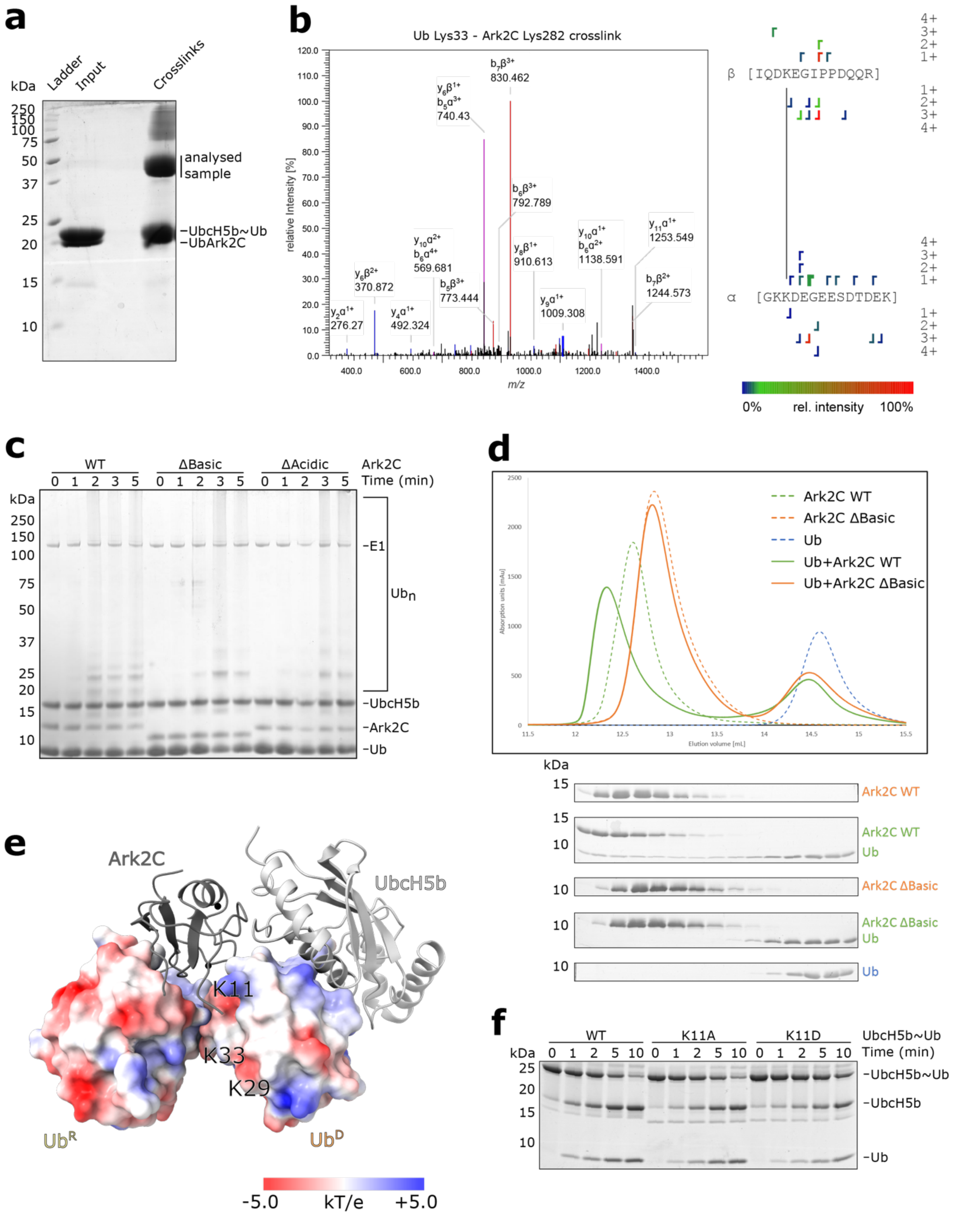

Supplementary Fig. 5 *The β3-RING loop contacts ubiquitin and enhances ubiquitin transfer.* (a) A 1:1 UbArk2C-UbcH5b~Ub complex (50 µM) was incubated with BS3-d0 (2 mM) crosslinker for 45 minutes and then resolved by SDS-PAGE. (b) Analysis of the excised band revealed a cross-link between Lys33 in ubiquitin and Lys282 in the loop. MS/MS spectrum of

the BS3-d0 cross-linked peptide with relevant b and y fragment ions highlighted on the spectrum, and the peptide sequence. The colour of the fragment marker indicates its relative intensity. (c) Multiturnover assays assessing the ability of the loop deletion (ΔAcidic and ΔBasic) mutants to assemble ubiquitin chains. Samples were resolved by SDS-PAGE and the gel was stained with Coomassie brilliant blue. (d) Analytical SEC analysis of Ark2C (WT and ΔBasic) in the presence and absence of ubiquitin. The chromatograms are shown in the top panel, with individual proteins shown as dashed lines and mixes as solid lines as indicated. Samples from each run (elution volume 12.0-15.0 mL) were resolved by SDS-PAGE and the gels were stained with Coomassie brilliant blue. (e) UbcH5b and Ark2C are represented as grey cartoons. Ub^R and Ub^D are shown as surfaces and are coloured according to electrostatic potential with contours at ±5.0 kT/e. The position of relevant residues in Ub^D is highlighted. (f) Discharge assays showing release of ubiquitin from the UbcH5b~Ub conjugates, with mutations as indicated. For the assay 0.75 µM of UbArk2C and 1 mM L-Lysine was used. The gel was stained with Coomassie brilliant blue.

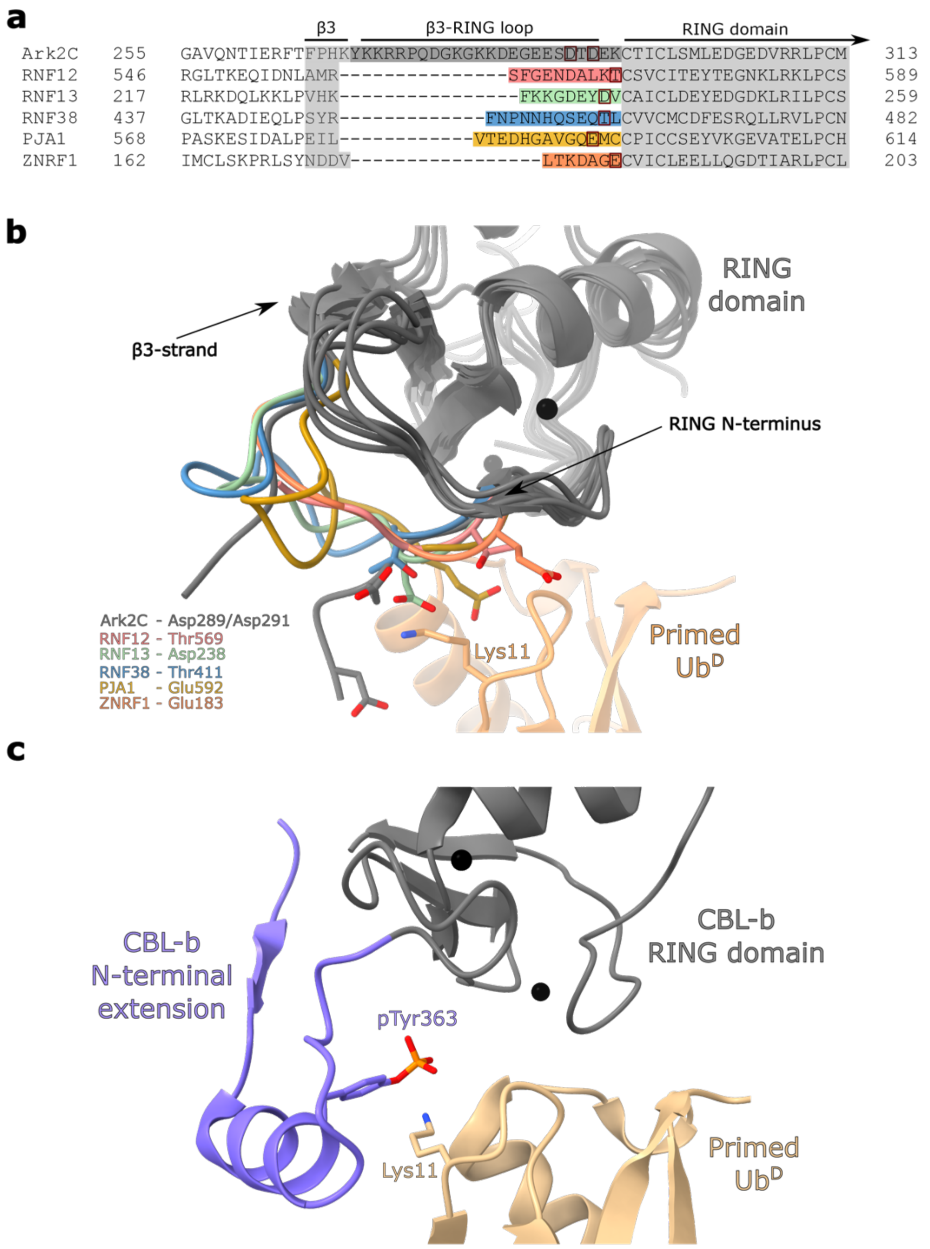

Supplementary Fig. 6 *The β3-RING loop is present in other monomeric RING-E3s.* (a) Sequence alignment of monomeric RINGs highlighting the β3-RING loop and the location of acidic and hydrophilic residues N-terminal to the core RING domain. (b) The RING domains of Ark2C (from the activated UbArk2C-UbcH5b~Ub complex), RNF12 (PDB: 6W9D), RNF13 (PDB: 5ZBU), RNF38 (4V3L), PJA1 (PDB: 2L0B), and ZNRF1 (PDB: 5YWR) were overlayed. Individual β3-RING loop colours correspond to their labels. Lys11 from Ub^D is shown in sticks. Black spheres represent zinc ions. (c) Contacts between CBL-b (grey) and Ub^D (orange) (PDB: 3ZNI). N-terminal extension of the CBL-b RING domain is shown as purple cartoon with pTyr363 in sticks. Lys11 from Ub^D is shown in sticks.
