# Supplementary material for: Ubiquitin and a charged loop regulate the ubiquitin E3 ligase activity of Ark2C
Source: Nat Commun. 2022 Mar 4;13:1181. doi: 10.1038/s41467-022-28782-y (PMC8897509; doi:10.1038/s41467-022-28782-y)
Supplement: Supplementary file 3 — Reporting Summary [file 41467_2022_28782_MOESM3_ESM.pdf]

Corresponding author(s): Catherine Day
Last updated by author(s): Jan 11, 2022

Reporting Summary

Nature Portfolio wishes to improve the reproducibility of the work that we publish. This form provides structure for consistency and transparency in reporting. For further information on Nature Portfolio policies, see our Editorial Policies and the Editorial Policy Checklist.

Statistics

For all statistical analyses, confirm that the following items are present in the figure legend, table legend, main text, or Methods section.

n/a	Confirmed	
☐	☒	The exact sample size (*n*) for each experimental group/condition, given as a discrete number and unit of measurement
☐	☒	A statement on whether measurements were taken from distinct samples or whether the same sample was measured repeatedly
☒	☐	The statistical test(s) used AND whether they are one- or two-sided<br>*Only common tests should be described solely by name; describe more complex techniques in the Methods section.*
☒	☐	A description of all covariates tested
☒	☐	A description of any assumptions or corrections, such as tests of normality and adjustment for multiple comparisons
☒	☐	A full description of the statistical parameters including central tendency (e.g. means) or other basic estimates (e.g. regression coefficient) AND variation (e.g. standard deviation) or associated estimates of uncertainty (e.g. confidence intervals)
☒	☐	For null hypothesis testing, the test statistic (e.g. *F*, *t*, *r*) with confidence intervals, effect sizes, degrees of freedom and *P* value noted<br>*Give P values as exact values whenever suitable.*
☒	☐	For Bayesian analysis, information on the choice of priors and Markov chain Monte Carlo settings
☒	☐	For hierarchical and complex designs, identification of the appropriate level for tests and full reporting of outcomes
☒	☐	Estimates of effect sizes (e.g. Cohen's *d*, Pearson's *r*), indicating how they were calculated

Our web collection on statistics for biologists contains articles on many of the points above.

Software and code

Policy information about availability of computer code

Data collection	XDS (BUILT=20200417), Unicorn (v7.0)
Data analysis	aimless (v0.6.2), PhaserMR (v2.8.2), Phenix.refine (v1.13_2998), Coot (v0.8.9), Pisa (v2.1.1), Inkscape (v1.1), USCF ChimeraX (v1.2.5), ImageStudio Lite (v5.2.5), StavroX (v2.0.1.4)

For manuscripts utilizing custom algorithms or software that are central to the research but not yet described in published literature, software must be made available to editors and reviewers. We strongly encourage code deposition in a community repository (e.g. GitHub). See the Nature Portfolio guidelines for submitting code & software for further information.

Data

Policy information about availability of data

All manuscripts must include a data availability statement. This statement should provide the following information, where applicable:

- Accession codes, unique identifiers, or web links for publicly available datasets
- A description of any restrictions on data availability
- For clinical datasets or third party data, please ensure that the statement adheres to our policy

The atomic coordinates and structure factors have been deposited in the Protein Data Bank with accession codes of 7R70 (UbArk2C fusion) and 7R71 (UbArk2C-UbcH5b~Ub complex). All data supporting the findings in this study are available within the article and in the source data file provided with this paper. Bespoke reagents are available from the corresponding author upon reasonable request.

Field-specific reporting

Please select the one below that is the best fit for your research. If you are not sure, read the appropriate sections before making your selection.

☒ Life sciences ☐ Behavioural & social sciences ☐ Ecological, evolutionary & environmental sciences

For a reference copy of the document with all sections, see nature.com/documents/nr-reporting-summary-flat.pdf

Life sciences study design

All studies must disclose on these points even when the disclosure is negative.

Sample size	X-ray data were collected on single crystals until acceptable completeness and redundancy was reached. No statistical methods were to predetermine the sample size, but experiments were repeated to verify the results. Samples sizes are specified in the legends.
Data exclusions	X-ray reflections were truncated based on CC1/2 statistics and <(I)/(sigma I)>. No other data were excluded.
Replication	All biochemical assays were replicated multiple times. The numbers of replicates are indicated in figure legends for quantified data, while other experiments were done at least twice. A statement has been added to the Methods section.
Randomization	No data randomisation was applied because all data were generated using recombinant proteins.
Blinding	Blinding was not necessary for in vitro experiments as there are no experiments where investigator bias could affect measurements or data analysis.

Reporting for specific materials, systems and methods

We require information from authors about some types of materials, experimental systems and methods used in many studies. Here, indicate whether each material, system or method listed is relevant to your study. If you are not sure if a list item applies to your research, read the appropriate section before selecting a response.

Materials & experimental systems

n/a	Involved in the study	
☒	☐	Antibodies
☒	☐	Eukaryotic cell lines
☒	☐	Palaeontology and archaeology
☒	☐	Animals and other organisms
☒	☐	Human research participants
☒	☐	Clinical data
☒	☐	Dual use research of concern

Methods

n/a	Involved in the study	
☒	☐	ChIP-seq
☒	☐	Flow cytometry
☒	☐	MRI-based neuroimaging
